# Guiding osteogenesis of mesenchymal stem cells using carbon-based nanomaterials

**DOI:** 10.1186/s40580-017-0096-z

**Published:** 2017-01-25

**Authors:** Ee-Seul Kang, Da-Seul Kim, Intan Rosalina Suhito, Sung-Sik Choo, Seung-Jae Kim, Inbeom Song, Tae-Hyung Kim

**Affiliations:** School of Integrative Engineering, Chung-Ang University, 84 Heukseok-ro, Dongjak-gu, Seoul, 06974 Republic of Korea

**Keywords:** Stem Cell, Chitosan, Graphene Oxide, Mesenchymal Stem Cell, Osteogenic Differentiation

## Abstract

In the field of regenerative medicine, stem cells are highly promising due to their innate ability to generate multiple types of cells that could replace/repair damaged parts of human organs and tissues. It has been reported that both in vitro and in vivo function/survival of stem cells could significantly be improved by utilizing functional materials such as biodegradable polymers, metal composites, nanopatterns and nanohybrid particles. Of various biocompatible materials available for use in stem cell-based therapy and research, carbon-based materials—including fullerenes graphene/graphene oxide and carbon nanotubes—have been found to possess unique physicochemical characteristics that contribute to the effective guidance of stem cell differentiation into specific lineages. In this review, we discuss a number of previous reports that investigated the use of carbon-based materials to control stem cell behavior, with a particular focus on their immense potential to guide the osteogenesis of mesenchymal stem cells (MSCs). We hope that this review will provide information on the full potential of using various carbon-based materials in stem cell-mediated regenerative therapy, particularly for bone regeneration and repair.

## Introduction

Regenerative medicine seeks to find effective methods or tools to treat damaged tissue and organs by restoring their structure and function. Numerous approaches have been proven useful to regenerate damaged parts of the human body, such as insertion of cell-friendly biomaterials [1, 2], injection of growth factors/chemicals [3], autologous tissue engraftments, and stem cell treatments [4]. Of these, stem cell-mediated tissue repair is one of the most promising areas, and has received an extensive amount of attention for more than a decade [5].

Stem cells encompass all cells capable of generating multiple types of cells through differentiation, and they are known to be useful resources to develop regenerative therapies [6–9]. Stem cells are generally categorized into cells with pluripotency (e.g., embryonic stem cells, induced pluripotent stem cells), and cells with multipotency (e.g., mesenchymal, hematopoietic, neural stem cells) [10]. Despite the limited capability for differentiation of multipotent stem cells compared to that of pluripotent stem cells, multipotent cells are still considered to be promising candidates for use in regenerative therapies since they are easy to obtain without encountering ethical issues and, most importantly, are relatively free from teratoma formation/tumor development [11].

Mesenchymal stem cells (MSCs) are a type of multipotent stem cells that can be obtained from bone marrow, adipose tissue and even from dental tissue. MSCs are capable of generating a variety of cells, including chondrocytes (cartilage), osteocytes (bone), myocytes (muscle), adipocytes (fat) and neuronal cells [12]. Hence, it is obvious that the differentiation of MSCs should be controlled with precision to achieve certain types of cells prior to transplantation in order to exploit the potential of MSC-based therapies to its fullest. To this end, the most common method to induce the differentiation of MSCs is the treatment with medium containing multiple growth factors, proteins, and chemicals that have been proven to be highly effective in guiding MSC differentiation toward specific lineages. Specifically, factors for osteogenic differentiation, such as bone morphogenetic protein (BMP), basic fibroblasts, dexamethasone, vitamin D3, beta glycerophosphate, and ascorbic acid have been reported to convert MSCs into osteoblasts with high efficacy [13].

While traditional materials/molecules, known as ‘soluble cues’ continue to show its effectiveness in MSC differentiation, other ‘insoluble cues’ have been reported to successfully regulate physical/mechanical properties of substrates in which cells attach, grow, and differentiate [14]. Micropatterns, nanopatterns, nanoparticles and bio-hybrid materials have been widely used as physicochemical factors in conjunction with proper functionalities on material surfaces in order to (i) maintain multipotency of the MSCs for long-term culture [15], (ii) control cell adhesion, migration and proliferation [16, 17], and (iii) guide their differentiation into specific lineages [1, 18, 19]. Among various materials available for stem cell studies, carbon-based materials, including fullerenes, carbon nanotubes (CNTs), and graphene/graphene oxide (GO) have shown immense potential for cell-friendly and cell-adhesive materials with lower toxicity [20, 21]. Remarkably, these carbon-based materials have demonstrated their excellent capabilities in stem cell differentiation into specific lineages, especially for bone (osteogenic) differentiation. This could be attributed to their unique surface properties, such as absorption/repulsion of specific differentiation factors, and improvement in cell adhesion via the interaction between the cell membrane and the surface of the carbon materials [22, 23].

**Fig. 1:**
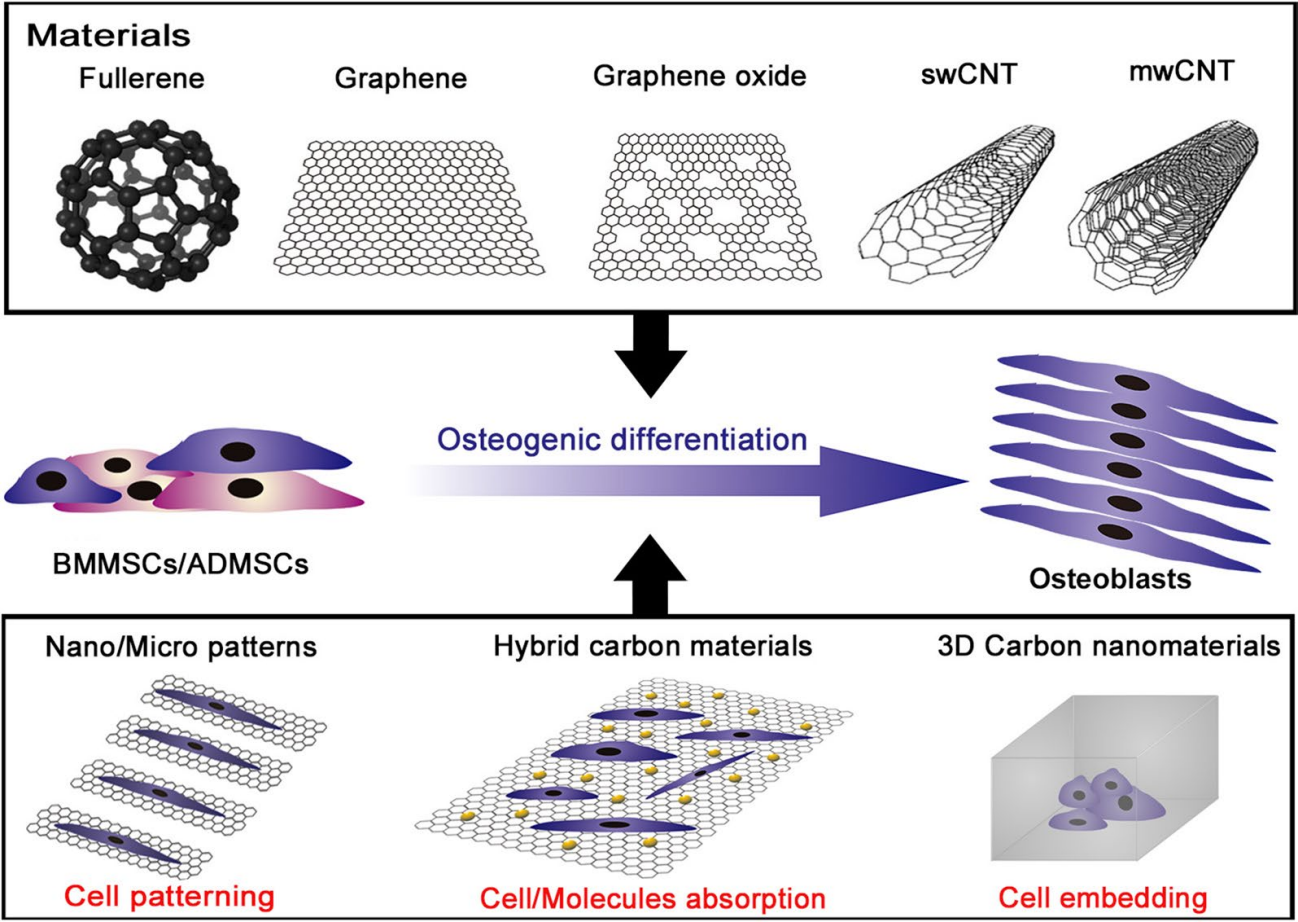
Schematic diagram depicting the guidance of osteogenesis for mesenchymal stem cells using carbon-based nanomaterials

Despite the physicochemical and mechanical characteristics of carbon-based materials to induce stem cell differentiation into specific lineages, there have been no reviews to date which explore specific application of carbon-based materials in osteogenic differentiation. To this end, this review will highlight and discuss various strategies in biomedical application of carbon-based materials, with a particular focus on their usage in osteogenic differentiation of MSCs, obtained from various tissues. In this study, carbon-based materials are categorized into three different groups: (i) fullerenes (zero dimensional material), (ii) graphene and GO (two dimensional material), and (iii) carbon nanotubes (three dimensional material). Thereafter, their mechanism of action (e.g., cell patterning, adhesion control, molecule absorption) and potential applications in the osteogenic differentiation of MSCs will be extensively discussed (Fig. 1).

## Guiding osteogenic differentiation of MSCs using pure carbon-based materials

### Fullerenes

Fullerenes, first designed by Kroto et al. [24], are molecules, consisted of carbon atoms (C_60_), that can be shaped into hollow spheres, spheroids, and numerous other forms. Their hollow shape is analogous to clathrin-coated vesicles in cells which allows fullerenes to be used as drug delivery vehicles or nano-sized gene-carriers. However, fullerene is also known to have high reactivity, attributed to its high angle strain, which could result in several detrimental effects on cells, such as denaturation of DNA or plasma membrane damage. Nonetheless, fullerenes could be employed to target tumor cells or antigens that are known to show resistance to certain medicine. Subsequently, the following section will explore the viability of fullerenes in biomedical application and regenerative medicine.

The first paper, describing the use of fullerenes in osteogenesis, was published by Bacakova et al. in 2007 [21]. The study initially explored the possibility of applying various types of carbon materials, such as fullerenes, carbon nanotubes, diamond layers, and carbon nanomaterials to bone tissue regeneration. Later on, the same research group also successfully synthesized fullerene layers, coated with carbon fiber-reinforced carbon composites (CFRC), and cultured MG 63 cells on a terpolymer of poly-tetrafluoroethylene. The nano-patterns, engraved on the CFRC surface, reportedly assimilated the extracellular environment that envelops the natural bone tissue, and consequently improved the adhesion and differentiation of bone-derived cells. The paper comprehensively suggested the possibility of exploiting fullerene layers for bone tissue regeneration of respective osteogenic cells.

On the other hand, Kopova et al. [25] also reported the effect of fullerenes on human bone-derived cells by using fresh (1 week old) and aged (1 year old) fullerene films.

**Fig. 2:**
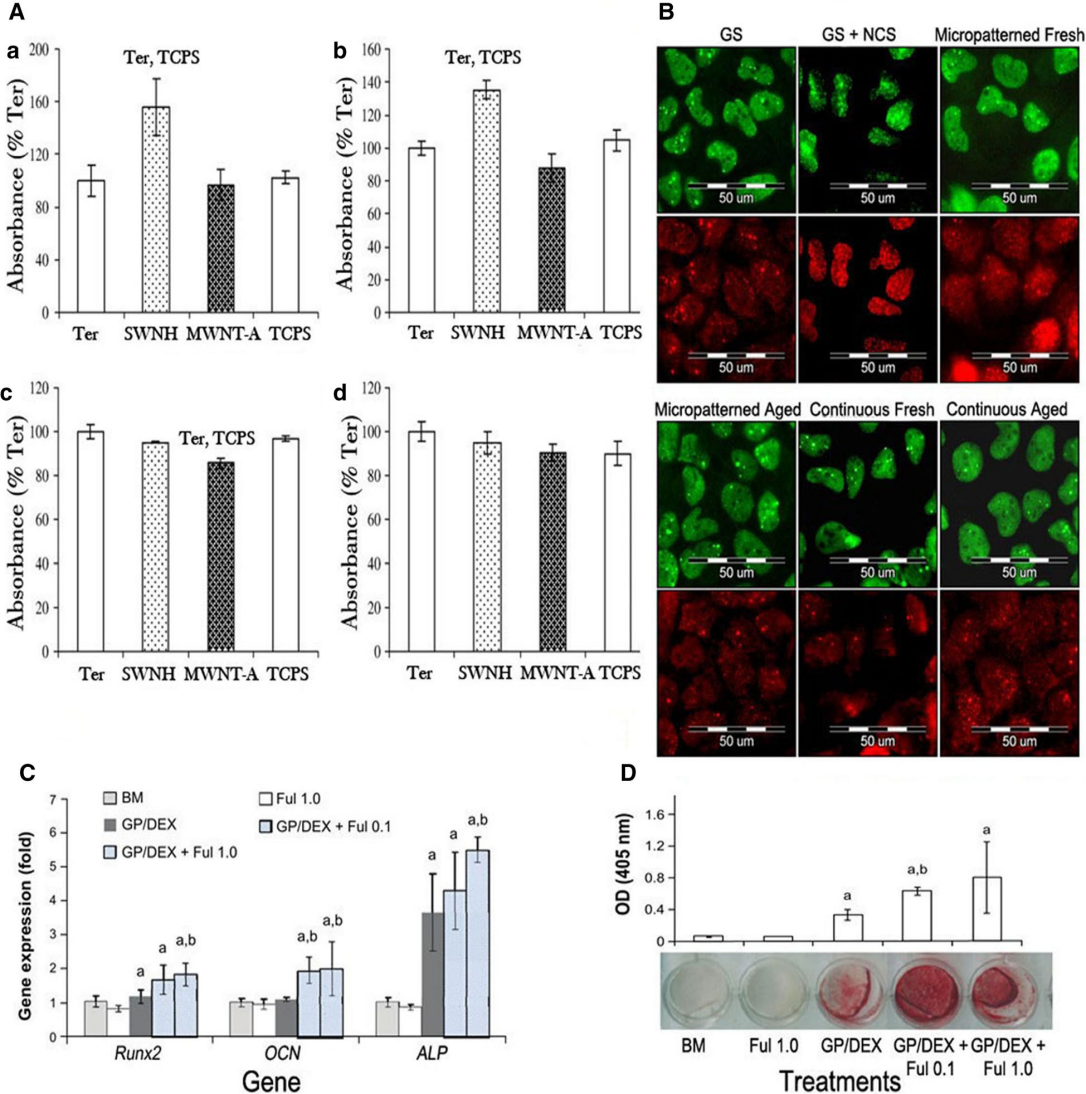
**A** Concentration of vinculin (*a*), talin (*b*), osteocalcin (*c*) and ICAM-1 (*d*) in osteoblast-like MG 63 cells on day 8 after seeding on the pure terpolymer of polytetrafluoroethylene, polyvinyldifluoride and polypropylene (Ter), terpolymer mixed with 4 wt% of single-wall carbon nanohorns (SWNH) or 4 wt% of high crystalline electric arc multi-wall carbon nanotubes (MWNT-A) and tissue culture polystyrene (TCPS). **B** Immunofluorescence staining of markers of DNA damage response: 53BP1 (*green*) and gamma-H2AX (*red*) in human osteoblast-like U-2 OS cells on micropatterned or continuous fresh and aged fullerene films after 7 days of cultivation. GS, microscopic glass coverslips, reference material; GS + NCS, positive control to DNA damage response, induced by 1 h incubation of U-2 OS cells in neocarzinostain (NCS; 700 ng/mL). Fullerol pre-treatment increased osteogenic potential of human aDscs. Cells were pretreated with fullerol (1.0 or 0.1 µM) for 7 days, followed by osteogenic induction for 14 days (n = 4). **C** Gene expression of osteogenic markers, Runx2, OCN, and ALP by real-time rT-Pcr at day 7, using 18 s as internal control. **D** Alizarin red staining at day 21. *Letters a* and *b* denote p = 0.05 versus BM and gP/DeX group, respectively. **A** The reprint of this figure from [21] is permitted by the Elsevier. **B** The reprint of this figure from [25] is permitted by MDPI **C, D** The re-print of this figure from [26] is permitted by Dove Medical Press

Kopova et al. used immunofluorescence staining on the two fullerene films to determine the amount of DNA damage on genes that control the growth of human bone tissue (Fig. 2). The staining results showed that cells cultured on fresh fullerene films showed a low level of activity similar to that of the control cells cultured on pure glass. However, the cell activity on the aged fullerene films was reported to be higher than that of cells grown on the fresh films. Therefore, it was concluded that the aged films offer more possibilities in culturing cells.

In 2014, Yang et al. [26] reported on the use of supporting materials for osteogenesis of human adipose-derived stem cells (hADSCs). The authors utilized antioxidant, hydroxylated fullerene (fullerol), and FoxO1 (transcription factor) to reduce cellular reactive oxygen species (ROS) activities, and induce osteoblast differentiation. Their results indicated minimal damage of human ADSCs by fullerene. Moreover, they found that nano-fullerols in fact increased human ADSC osteogenesis by procreating a low intracellular ROS level for the cells during the bone formation. Lastly, they also suggested a mechanism to regulate osteoblast differentiation of MSCs by elevating FoxO1 and reducing the ROS.

Likewise, Kopova et al. [27] reported on two different types of fullerene/Ti films and their effects on the growth and potential damage of human bone cells. Kopova and his team successfully synthesized composites, infused with a micro-patterned complex by mixing fullerene and titanium. They compared the MG-63 cell activities on between pure fullerene films and C_60_/Ti films. It was revealed that the fullerene combined with titanium films, fresh films, and aged films showed no ostensive differences on cell growth, such as their metabolic activity or viability. Moreover, it was also deduced that there were no confirmed signs of DNA damage when cultivating cells on mixed films.

It could be conclusively postulated that fullerenes offer multitudinous possibilities in regenerative medicine and tissue engineering. Despite initial complications with cytotoxicity and its tendency to induce DNA damage, fullerenes could be minimized by either incorporating with other substances (titanium), or modifying its surface with chemicals. Moreover, its unique surface property that closely assimilates the bone tissue matrix environment would serve as a great unparalleled asset in future tissue engineering applications.

### Graphene and graphene oxide

While chitosan, poly-l-lactic acid (PLLA), and polycaprolactone (PCL) have been widely used for bone regeneration, challenges still remain in assimilating their mechanical and chemical properties to those of the natural bone tissue [28]. In addition, some polymers require chemical modifications, owing to their lack of sites for cell adhesion and frequent immune responses, triggered by their byproducts [29]. However, graphene and its derivatives [graphene oxide (GO), and reduced graphene oxide (rGO)] have all shown to induce and sustain stem cell growth and differentiation with considerable efficacy, while being capable of enhancing osteogenic differentiation [22]. Thus, graphene and its derivatives have emerged as the next promising material in bone regeneration and tissue engineering.

First, graphene is an allotrope of carbon molecules that comes in shapes of two-dimensional, atomic-scale, honey-comb lattice. It has several novel properties, including excellent mechanical strength, and high thermal and electrical conductivity, making it an apropos candidate for electro-convulsive therapy. Consequently, graphene has been used in electrical, electrochemical, and optical applications [30, 31] as a newly applicable material for stem cell applications, such as osteogenesis. It is commonly obtained via chemical vapor deposition (CVD), which is often used in high-quality and high-quantity production prior to transfer into various substrates [32]. Considering the fact that graphene is non-cytotoxic and allows mesenchymal stem cells to attach and proliferate [33, 34], it has become an increasingly promising substrate for anchorage-dependent cells, i.e., mesenchymal stem cells (MSCs) since anchorage-dependent cells must adhere to substrates in order to spread, proliferate, and function properly [20, 35]. However, carbon-based materials exhibit different effects on cells when administered in vivo since they present various bio-distribution patterns [36, 37]. In that sense, graphene can be functionalized to develop bioactivity of the composite, or to be used as a surface coating on biomaterial substrates. This arises from the surface chemistry of biomaterials which serve as a potential tool to control biological responses [38] while cell adhesion, viability, and proliferation determine the biocompatibility of the substrate [20, 32, 35–39].

**Fig. 3:**
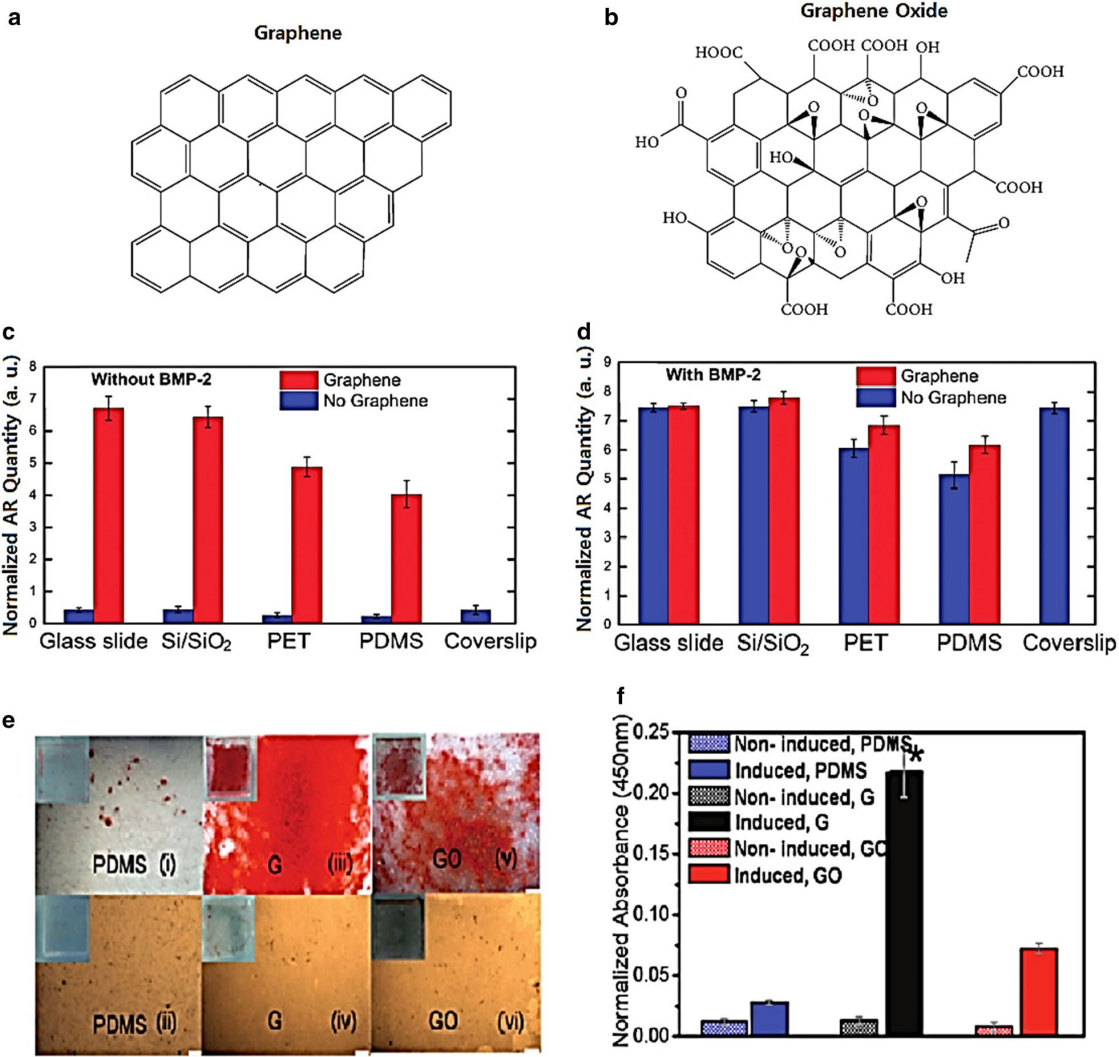
**A** Shape of graphene, **B** shape of graphene oxide. **C** Cells grown in the absence of BMP-2. Control with coverslips is shown as a reference. **D** Cells grown in the presence of BMP-2. Conventional plain coverslips were used as a positive control. **E** Osteogenic differentiation visualized by Alizarin Red staining after 12 days of incubation, on PDMS (*i*) with induction and (*ii*) without induction, on G (*iii*) with induction and (*iv*) without induction, and on GO (*v*) with induction (*vi*) and without induction. *Scale bars* are 200 μm. **F** The quantification demonstrated a significantly higher amount of Alizarin Red staining in the MSCs differentiated on G (*p < 0.05; n = 4 for each group) (A, B) The reprint of this figure from [22] is permitted by the Hindawi Publishing Corporation (C, D) The reprint of this figure from [42] is permitted by the American Chemical Society. (E, F) The reprint of this figure from [13] is permitted by the American Chemical Society

Several studies have found that graphene has an ability to capture stem cells [40] and control osteogenic differentiation of human mesenchymal stem cells (hMSC) [41]. Nayak et al. [42] found that graphene induces osteogenesis when cultured without BMP-2 (common growth factor for bone generation). The authors used four different substrates (polydimethylsiloxane (PDMS), PET, glass slide and Si/SiO_2_) with varying stiffness and surface roughness to investigate the effect of graphene on stem cell growth. As shown in Fig. 3C, D, when cultured without BMP-2, all four substrates with graphene showed a higher quantity of alizarin red compared to substrates without graphene. In addition, even after being cultured with BMP-2, the groups with graphene showed a higher concentration of alizarin red. This proved that graphene not only shows a tendency for osteogenesis of hMSCs, but also a synergetic effect when used with BMP-2 for bone tissue generation.

Moreover, Lee et al. [13] reported that the binding ability of graphene accelerates the differentiation of MSCs toward the osteogenic lineage. They conducted an experiment in which they cultured MSCs on graphene made via CVD. During the culture process, they introduced certain chemicals, such as insulin, dexamethasone, β-glycerolphosphate, and ascorbic acid. As illustrated in Fig. 3E, F, graphene has a tendency to pre-concentrate osteogenic inducers which consequently enhance the osteogenic differentiation of hMSCs. While prior studies have shown that it took 21 days to complete chemically-induced osteogenic differentiation [43], Lee’s study showed that it only took 12 days using graphene.

GO is a form of graphene that includes numerous oxygen atoms. Its oxygen composition gives rise to various functional groups in GO, such as epoxide, carboxyl, and hydroxyl groups [44]. These functional groups provide GO a greater mechanical strength, and allow it to interact with polymers more actively than non-functionalized graphene [13]. Rameshwar et al. showed synergetic effect of GO with an osteoinductive material [45]. Rameshwar’s team used calcium phosphates (CaP) as a biomaterial for their osteoconductivity and osteoinductivity. They synthesized GO-CaP nanocomposites and introduced them to hMSCs in osteogenic medium. The results from immunofluorescence staining of the osteoblast markers showed that the osteogenesis process was successful in the consecutive order of GO-CaP, CaP, GO, and control. Their research conclusively showed that GO-CaP nanocomposites not only facilitated the osteogenesis of hMSCs, but also enhanced calcium deposition by the osteoblasts. Hence, we conclude that graphene and GO both enhance and control the osteogenic differentiation of hMSCs.

**Fig. 4:**
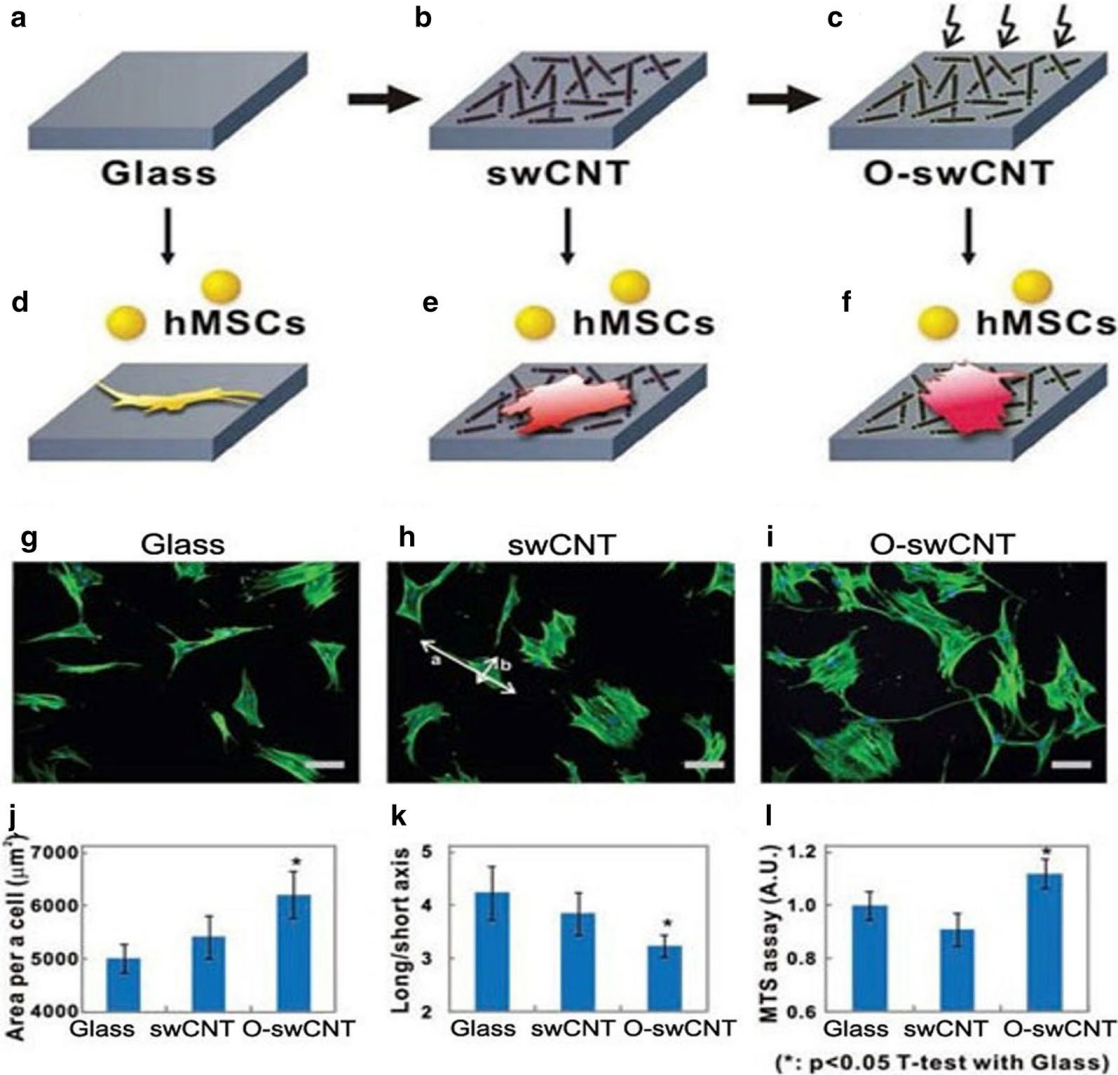
hMSCs growth on swCNT monolayers. **A** Glass substrate was used as a control. **B** swCNTs were adsorbed onto the glass substrate to form a swCNT monolayer. **C** Oxygen plasma treatment was applied to modulate the swCNT surface properties. Adhesion and proliferation of hMSCs on various substrates. Fluorescence images of actin filaments show the morphology of hMSCs on **D** a glass substrate, **E** a swCNT monolayer, and **F** an oxygen-plasma-treated swCNT monolayer (O-swCNT). *Scale bars* are 100 μm. The quantitative analysis was visualized with **g** the averaged value of area per cell (number of cells, *n* = 200), **H** the averaged value of the ratio of long and short axial lengths (*a*/*b* in **b**; *n* = 200), and **I** the averaged MTS assay value at day 6 (*n* = 3). In all analyses, Student’s *t* test was utilized to calculate the significance (*p < 0.05). Reprinted with permission from [14]. The reprint of this figure from [56] is permitted by WILEY–VCH Verlag GmbH & Co

### Carbon nanotubes

Carbon nanotubes (CNTs) are another type of carbon-based nanomaterials that are known to support the attachment, and growth of adult stem cells and progenitor cells, including osteoblasts and myoblasts [46, 47]. They are composed of rolled up nano-sized graphene sheets with a helical structure and π electron conjugation which provide elasticity, high chemical stability, and electrical conductivity [48, 49]. CNTs’ unique properties attribute to its versatility to be used in various areas, including nanotechnology, material science, and electronics. In recent studies, CNT-based materials were found to have considerable potentials for their use in numerous cellular applications, including cancer therapy [50] and tissue engineering [51]. Subsequently, their ability to support cell adhesion, cell growth, and differentiation has been considered for use with various stem cell lines, including hMSCs, neural stem cells, and embryonic stem cells [52–55].

CNTs are generally classified into single-walled CNT (swCNT), multi-walled CNT (mwCNT), and functionalized CNT, all of which can be easily adjusted to increase their biocompatibility as a cellular culture substrate. It is reported that swCNTs can promote the differentiation of hMSCs without the need for differentiation-inducing media [56]. Few studies have shown that hMSCs formed focal adhesions and grew in a considerable rate on single-walled CNTs (swCNTs) [46, 54]. Moreover, swCNTs treated with oxygen plasma have shown synergetic effects on differentiation and adhesion of the hMSCs [56]. The stress from stretching stem cells on microscale molecular patterns generates tension on the actin filaments, which in return enhances osteogenesis [57].

**Fig. 5:**
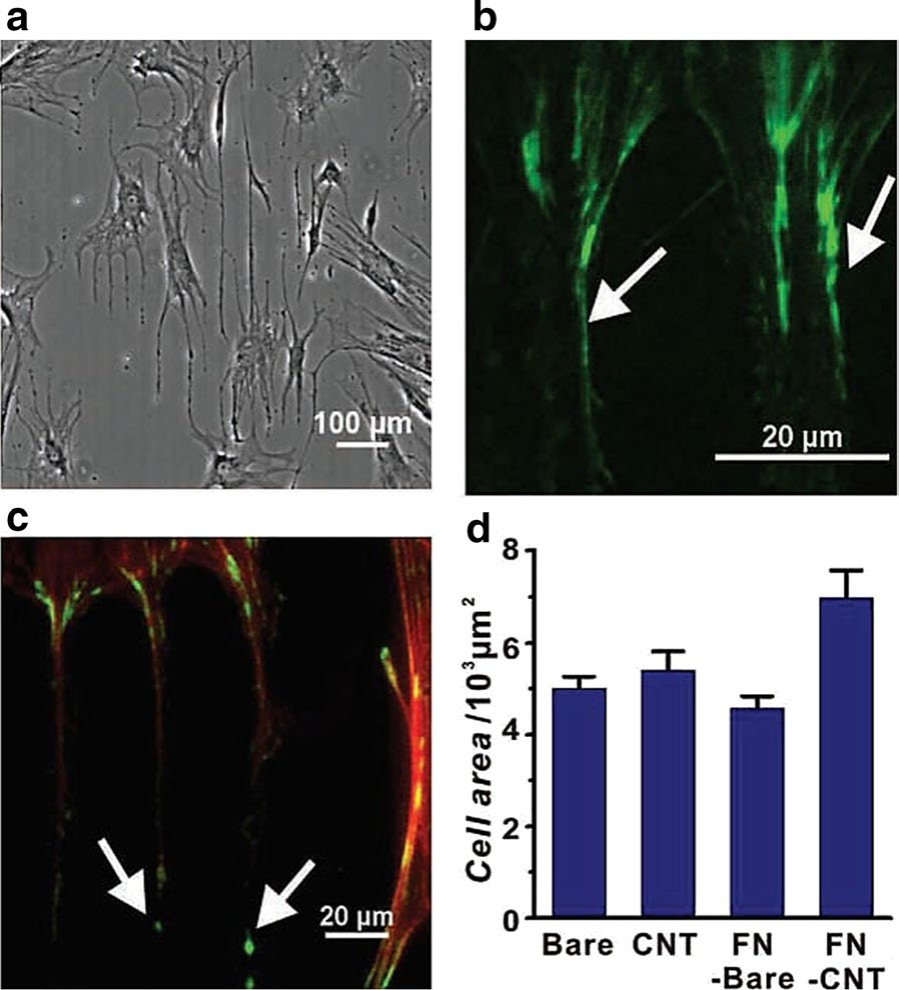
Selective growth of hMSCs on FN-CNT nanostructures. **A** On the glass substrates, the phase-contrast image of hMSCs on line patterns of FN-CNT structures without swCNTs. **B** Immunostained by vinculins (*green*) of the hMSCs. Vinculins are the full of edge of FN-CNT structure. **C** Fluorescence image of actin (*red*) and vinculins (*green*) of the hMSCs. Also grown on bare-glass. Arrows are focal-adhesion sites. **D** Average result of the cell area on bare glass, CNT, FN coated bare glass, swCNT-coated glass treated FNs. The reprint of this figure from [59] is permitted by WILEY–VCH Verlag GmbH & Co

Baik et al. [56] conducted an experiment where each of pure swCNTs and oxygen-plasma treated swCNT (O-swCNT) monolayers on a glass surface (Fig. 4B,C) were used as substrates with glass as control (Fig. 4A). The MSCs for osteogenic differentiation were seeded onto the substrates, and the cell adhesion process was analyzed by staining the actin fibers which indicated the average area per cell. Cell adhesion and proliferation of hMSCs were visualized by culturing hMSCs on each of the substrates (Figs. 4D-I, 5).

Zhang et al. [58] reported that the nanoscale surface roughness of swCNTs affects the deformation of the cell membrane and impacts distribution and diffusion of membrane proteins, including the focal adhesion proteins that are crucial in cell adhesion. It was concluded that the effect of swCNTs on adhesion proteins promotes hMSCs and osteoblast-like-cells to grow better on swCNT films than on glass substrates. It was also reported that CNTs encompass the ability to improve the absorption of extracellular matrix (ECM) proteins, i.e. fibronectin (FN), laminin, and vitronectin [59]. FN-CNT hybrid nanostructures have shown to improve either the cell adhesion or cell growth more efficiently than glass substrates (Fig. 5). For instance, FN-coated swCNTs were shown to improve the spreading of hMSCs more efficiently than common tissue culture plates [54], while improvement in induction of osteogenesis of hMSC was also observed using msCNTs. However, the same degree of improvement was not observed with the use of graphite due to the diversity in topography, and the proteins secreted from the cultured cells [60, 61]. It was concluded that the topography of CNTs exerts significant effects on cell spreading, morphology and different stem cell lineages [19, 62–65].

**Fig. 6:**
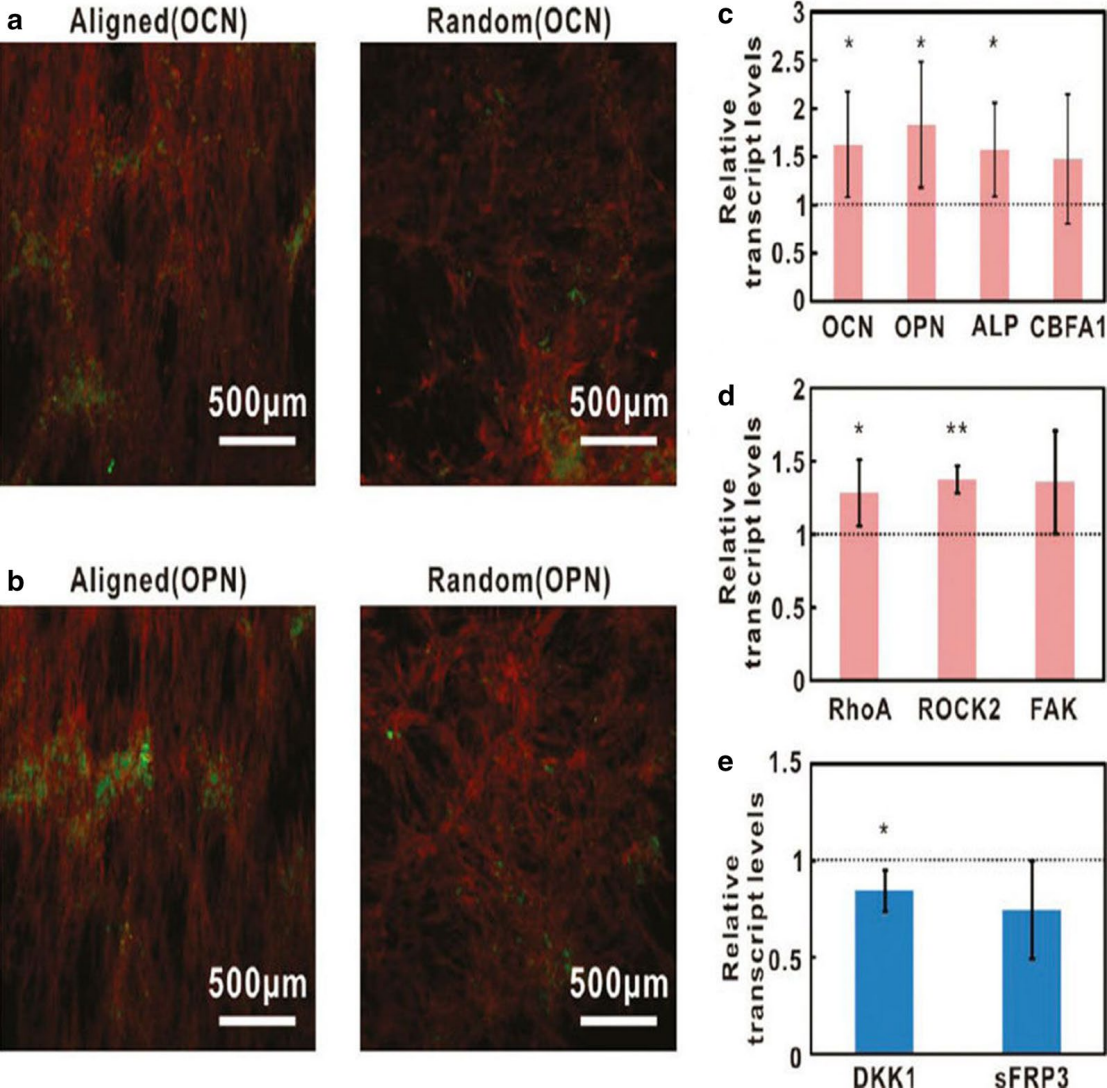
Osteogenic differentiation of hMSCs on two forms of CNT platform (**A**, **B**). Immunofluorescence images of osteocalcins (OCNs, *green*) and osteopontin (OPN, *green*) respectively, in hMSCs. The actin of hMSCs was stained with TRITC-phalloidins (*red*). **C** Expression levels of osteogenic genes, such as OCN, OPN, alkaline phosphatase (ALP), and core binding factor or alpha1 (CBFA1). **D** Expression levels of genes included transduction pathways such as focal adhesion kinase (FAK), Rho family of GTPases member A (RhoA), and Rho-associated coiled-coil protein kinas (ROCK). **E** Expression level of the genes of Wnt-antagonists, such as dikkopf-1 (DKK1) and secreted frizzled-related protein type 3 (sFRP3). The reprint of this figure from [66] is permitted by the American Chemical Society

On the other hand, square-patterned and ranged CNTs can also be used to increase the expression of particular osteogenic genes, such as osteocalcin and alkaline phosphatase [56]. The modified topography of CNTs enhanced both cell proliferation and osteogenic differentiation of hMSCs while the alignment of CNTs improved the expression of the osteoblast gene [66], and the hMSC media was dispersed more easily by functionalized CNTs (Fig. 6).

Despite numerous advantages that CNTs present, several cases of in vivo toxicity have been reported. The most noticeable toxicity arises from DNA destruction in mouse embryonic stem cells (mESCs), due to the increased oxidative stress. Consequently, the rise in oxidative stress generates free radicals, which leads to increased peroxidative condition [67, 68]. Although it was shown that COOH- functionalized CNTs have less in vivo cytotoxicity [31], CNTs are hardly used seperately for medical purposes without modification of their surface properties. There had been efforts to increase the activity and interaction of CNTs through surface modification [69], however the toxicity of CNTs remains to be a persistent problem in biomedical application.

Therefore, CNTs are considered to be a promising biomedical material with multitudinous potentials as they can serve as representatives of microenvironment and nanotopography. Despite several complications concerning oxidative cytotoxicity, CNTs will continue to be explored for its application in biomedical engineering and tissue therapies.

## Guiding osteogenic differentiation of MSCs using modified carbon-based materials

### Nano/micro patterns of carbon nanomaterials

It is commonly known that changes in the microenvironment induce dynamic differentiation of hMSCs. While nano/micro patterns have been reported to be highly effective in modulating stem cell differentiation by regulating the cell affinity and extension, carbon-derived patterns are also capable of upregulating the differentiation of hMSCs. In that sense, patterned graphene was tested for its capacity to differentiate hMSCs in comparison to efficacy of graphene in differentiation of hMSCs.

Kim et al. [70] reported that nano-sized Graphene Oxide (NGO) patterns, with a size approximately of 100 nm, enhanced the elongation of hMSCs, and resulted in a considerable increase in osteogenesis of hMSCs in comparison to common culture plates and polydimethylsiloxane (PDMS). Moreover, a graphene nanogrid, constructed from graphene nanoribbons (GONRs), which are elongated strips of graphene, was also reported to enhance adhesion and differentiation of hMSCs even without differentiation factors [71]. Similarly, Akhavan et al. [34] reported that the rate of proliferation of the MSCs on the nanogrid was higher than that of non-regulated proliferation on PDMS and GO sheets. Therefore, it could be concluded that the rGONR attributed to the absorption rate of differentiation chemicals, as well as the surface topography of the patterns.

**Fig. 7:**
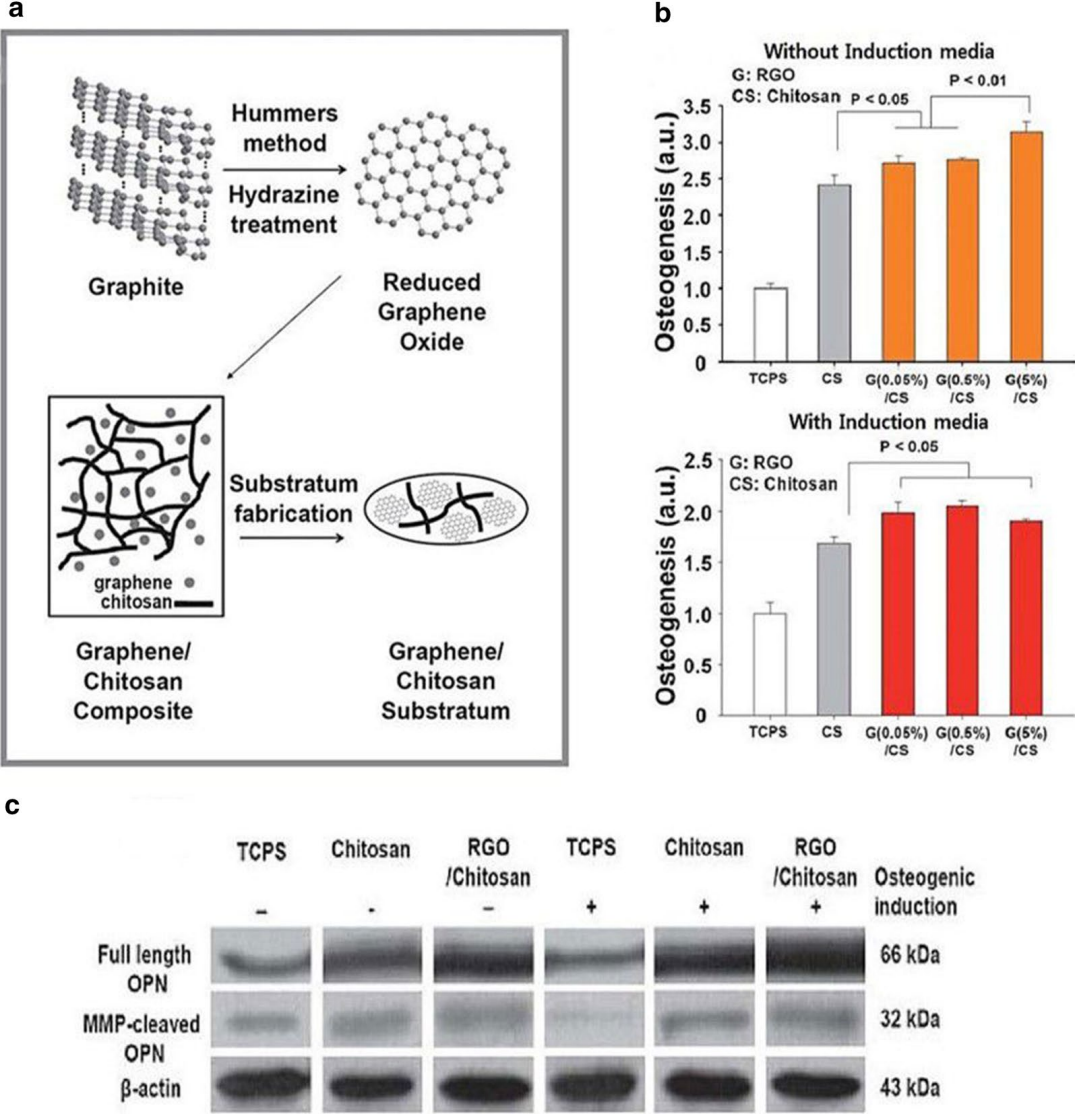
**A** Schematic diagram showing the synthesis of graphene-incorporated chitosan nanocomposite substrata. **B** Quantification of the degree of osteogenesis confirmed by the mineralization as measured by Alizarin Red S staining of hMSCs cultured on the RGO–chitosan substrata, chitosan substrata, and TCPS for 21 days. **C** Western blot analysis of full length OCN expression and expression of MMP-cleaved OPN in hMSCs cultured on 5% RGO–chitosan substrata, chitosan substrata, and TCPS. The reprint of this figure from [78] is permitted by the Royal Society of Chemistry

In addition, similar acceleration in cellular activities was also observed on CNTs with engraved nanopatterns. First, aligned CNTs showed a higher rate of proliferation and differentiation for the hMSCs than the CNTs that are randomly oriented as seen in Fig. 7 [78]. Namgung et al. [47] cultured hMSCs on the aligned CNT network as well as on the randomly oriented network to investigate whether the arrangement of CNTs would affect the transduction mechanism of hMSCs. Their results showed that the transduction mechanism, promoted by the high tension in the elongated hMSCs on the oriented CNTs, indeed promoted both proliferation and osteogenesis of hMSCs. Park et al. discovered that CNT/Nanowire (CNT/NW) monolayer patterns could also play an important role in controlling stem cell adhesion and morphology. Generally, MSC shows higher affinity to CNT patterns than the NW coating. This could be exploited to produce high density swCNT patterns on electrodes, allowing MSCs to grow on the swCNT patterns with substantial efficiency.

MSC differentiation is often influenced by the nanoscale diversity of swCNTs, including thickness, roughness and surface characteristics [72]. Lee et al. reported the thickness of their swCNTs to be about 95 nm with a roughness of 9.81 nm which reportedly accelerated the rat MSCs (rMSCs) growth. On the other hand, Bitirim et al. [73] informed that MSCs showed similar attachment tendencies to both collagen-coated swCNT plates, and those without collagen coating. However, the collagen-coated CNTs had a stronger adhesion of MSCs, which implies the importance of surface properties in the adhesion of MSCs. In addition, a case was reported where linear CNT patterns were able to control the morphology of hMSCs to differentiate into neural cells [74], which suggests that the pattern of carbon-derived material is excellent for the osteogenic differentiation of MSCs.

Therefore, it could be concluded that controlling the differentiation of hMSCs on the carbon material patterns offers multitudinous opportunities, especially in osteogenic differentiation and neural differentiation. However, further studies on both properties of carbon patterns and hMSCs are still necessary in order to overcome the cytotoxicity problem of CNTs. Nonetheless, the carbon material patterns show abounding potentials in regenerative engineering, as well as in biomedical and tissue therapies.

### Hybrid carbon nanomaterials

Graphene is known to have unique physicochemical properties which allows it to be considered for numerous integrative applications when utilized with other polymers. Numerous studies have investigated the viability of graphene-based hybrid nanomaterials, such as graphene polymer nanoparticles, nanoparticle-decorated graphene sheets, graphene-embedded nanoparticles, and graphene-encapsulated nanoparticles in assisting the cellular activity of various types of stem cells, including embryonic stem cells (ESCs), induced pluripotent stem cells (iPSCs), mesenchymal stem cells (MSCs), and neural stem cells (NSCs) [75, 76]. For instance, Solanki et al. [77] developed a new graphene-nanoparticle hybrid structure, encapsulated with GO on the surface of positively-charged silica nanoparticles (SiNP-GO), to enhance the differentiation of human neural stem cells (hNSCs). As a result, numerous studies have discussed the control and acceleration of osteogenic differentiation of hMSCs on graphene based hybrid nanomaterials.

As previously mentioned, graphene-based hybrid nanomaterials, such as graphene oxide (GO), have chemical, electrical, and mechanical properties favorable for tissue engineering applications. In 2011, Nayak et al. [42] reported that a graphene-coated substrate accelerated the osteogenesis of hMSCs. The authors showed that the hMSCs, incubated on graphene-coated Si/SiO_2_ substrates, showed accelerated differentiation, but the presence of graphene did not affect the morphology and growth of the cells in normal cell media compared to other substrates (PDMS, PET, glass slide).

Kim et al. [78] also demonstrated that graphene-coated surfaces are potentially valuable as conducive material for protein attachment due to the presence of hydrophobic and hydrophilic patches. They produced a graphene-based hybrid substrate, using a graphene-incorporated chitosan substrate (Fig. 7A). The substrates were fabricated after spin-coating rGO and chitosan mixtures (0, 0.05, 0.5, and 5%) on bare glass, ranging from 0.9, 1.5, 3.7, to 7.7 nm on average. Figure 7B shows the degree of hMSCs osteogenesis for 21 days on TCPS (as control) and the 0-5% RGO–chitosan substrata, as analyzed using Alizarin Red S staining, and measured with an ELISA reader.

The result showed that the osteogenic differentiation values of the hMSCs on the RGO–chitosan substrata were higher than those of the TCPS and chitosan substrate. In addition, Western blot analysis of hMSCs, cultured on 5% RGO–chitosan substrate, chitosan substrate, and TCPS, was conducted in the presence of osteocalcin (OCN) (Fig. 7C), an osteogenic gene, to confirm the improvement in osteogenesis. The results also showed that the OCN protein on the graphene–chitosan substrate was up-regulated compared to that on the TCPS and chitosan substrata. It was also reported that the MMP-cleaved osteopontin (MMP-cleaved OPN) led to a considerable increase in cell adhesion. Moreover, the protein expression of the MMP-cleaved OPN of hMSCs was analyzed, and the results indicated that the MMP-cleaved OPN on the RGO–chitosan substrate yielded more protein products than TCPS and chitosan substrate. In spite of the small amount of graphene used, these results indicated that the graphene-incorporated chitosan substrate could promote the osteogenesis of hMSCs.

Therefore, graphene-incorporated chitosan nanocomposites have shown immense potentials in enhancing the adhesion and differentiation of the hMSCs. The studies above delineated the versatile capability of graphene hybrid materials in differentiation of various types of stem cells, especially hMSCs. As such, graphene-based hybrid nanomaterials can be employed as part of an effective strategy in biomedical applications and stem cell tissue engineering systems [79].

### Three-dimensional carbon nanomaterials

Carbon nanomaterials come in forms of graphene sheets, ranging from zero-dimensional (0D) to two-dimensional (2D), and three-dimensional (3D) graphene structures [80]. While graphene is widely recognized for their unique physicochemical properties both separately and as hybrid carbon-based nanomaterials, the versatility of graphene has expanded to its application in three-dimensional structures. The following section will review information on three-dimensional graphene structures, such as graphene nano-onions (GNOs), graphene nanoribbons (GONRs), graphene nanoplatelets (GONPs), 3D graphene oxide encapsulated gold nanoparticle, and 3D graphene foams (GFs) [80–82]. The three-dimensional graphene structures are mostly utilized in detection of neural stem cell differentiation.

In 2013, Kim et al. proposed a spectroelectrochemical method to fabricate 3D graphene oxide encapsulated gold nanoparticles. The fabricated 3D GO structure was enhanced by combined chemical/electromagnetic enhancement of raman signals. In order to account for undifferentiated stem cells with high C=C saturation, the fabricated structure was merged with SERS, which was highly effective in monitoring neural stem cell differentiation.

Talukdar et al. on the other hand, reportedly provided morphological information on GNOs, GONRs, and GONPs with transmission electron microscope (TEM) [81]. The diameters of the GNOs were in the range from 50 to 300 nm, and GNOs came in shapes of onions with vacant, multi-walled, and concentric polyhedral structures. In addition, Talukdar found out that GONRs came in shapes of rectangular sheets, and GONPs had disk-like morphology.

**Fig. 8:**
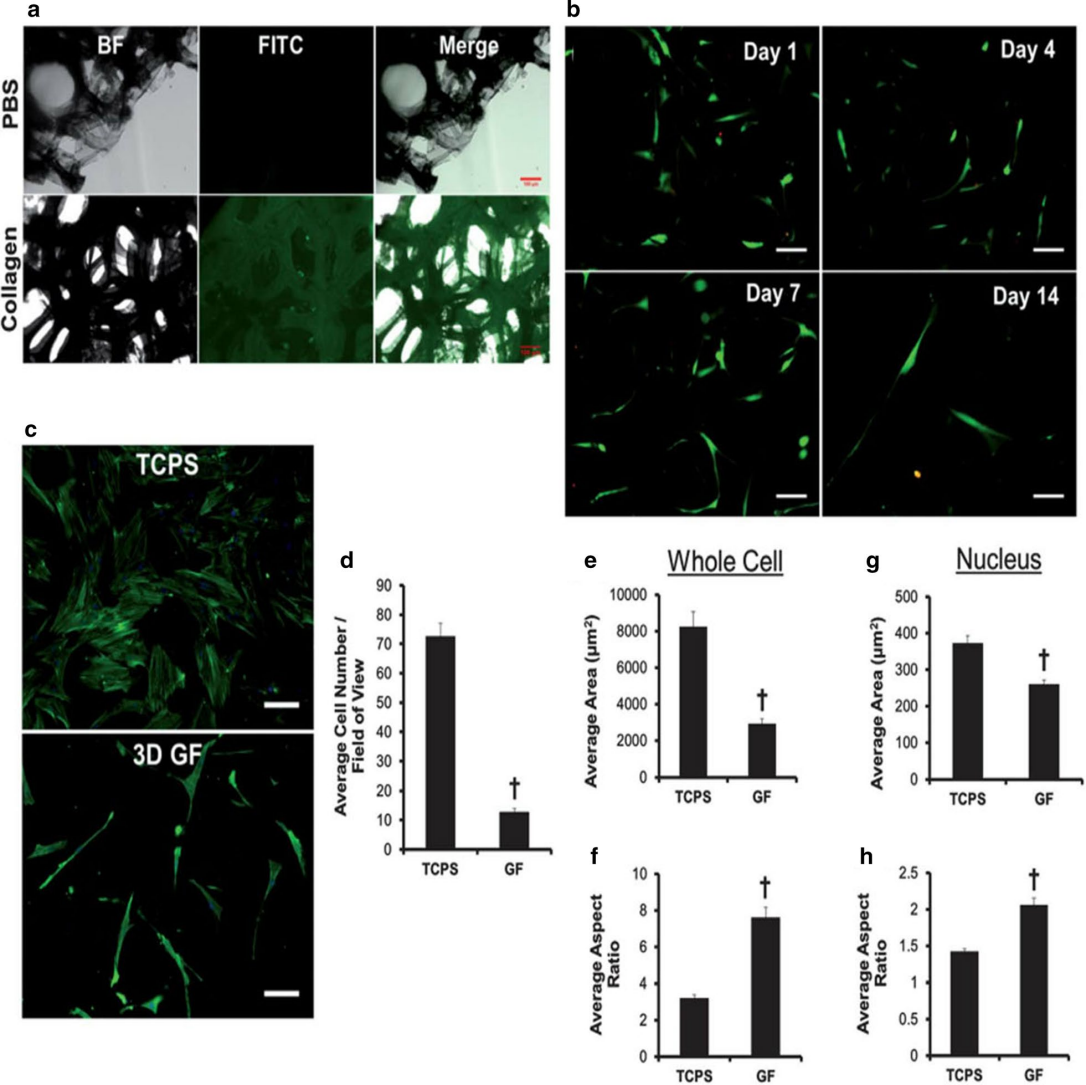
Protein adsorption, hMSC attachment, and morphological changes on 3D GFs. **A** Images of bright field (BF), fluorescent (FITC), and merged images reveal FITC-labeled collagen homogeneously adsorbed onto the surface of graphene foams (GFs). **B** The attachment and viability of FITC-labeled hMSCs were supported by GFs over 14 days. **C** FITC-labeled hMSCs were cultured in GFs and TCPS control over 7 days. **D** Quantification of FITC-labeled hMSCs were cultured in GFs and TCPS control over 7 days. **E** Quantification number of number of whole cell and **F** aspect ratio. **G** Quantitatively nuclear morphology and **H** aspect ratio. The re-print of this figure from [41] is permitted by the Royal Society of Chemistry

However, we wish to highlight the promotion of osteogenesis by hMSCs using graphene foams (GFs) substrate [80]. The GFs were initially grown on a 3D nickel scaffold after which Ni was removed by FeCl_3_ etching. As presented in Fig. 8A, hMSCs attachment was increased in the GF substrate, co-localized with collagen, one of the ECM proteins known to bind hMSCs, when compared to phosphate-buffered saline (PBS). Moreover, the cell viability was observed for the hMCS that was cultured in 3D GFs substrate for over 14 days (Fig. 8B). The results showed that most viable cells (green) survived with few dead cells (red), indicating that the GFs are capable of supporting the attachment of hMSCs, as well as cell viability. Moreover, the GFs substrate was shown for 7 days in comparison to TCPS to have fewer number of cells (Fig. 8C) which implies the radical difference in whole cell and nuclear morphologies. However, as shown in Fig. 8A-C, the hMSCs on the GFs showed long protrusions up to 100 mm in length. The authors claim that the high porosity of the material resulted in the lower hMSC attachment on the GFs than that on the TCPS. However, the reduced attachment could be improved by employing a bioreactor, and through pore architecture/size optimization.

Therefore, as demonstrated in the studies above, GFs has enough potentials to be utilized as an effective substrate to enhance the attachment of hMSCs, maintain the cell viability, and stimulate morphological changes without extrinsic biochemical inputs.

## Conclusion

In this study, we highlighted several studies utilizing carbon-based nanomaterials for biomedical applications, with particular focus on the use of fullerene, CNT, and graphene and its derivatives to guide the osteogenesis of MSCs.

A number of previous studies have confirmed that carbon-based materials and their related materials possess the ability to induce hMSC differentiation into specific lineages. Specifically, the use of carbon-based materials in combination with differentiation factors/osteoinductive agents (e.g. peptides, proteins, growth factors) have been reported. These materials were found to act as an attracting signal for the osteogenesis of MSCs, as well as for bone cells to promote the bone regeneration process. Among several carbon-based materials, graphene and graphene derivatives have shown great potential for stem cell research, due in part to their unique physicochemical properties such as high surface area, ease of functionalization, and low cytotoxicity. These properties are superior to other types of carbon-based materials in term of embedding drugs/growth factors and enhancing cell adhesion, proliferation, and differentiation of hMSCs into osteogenic lineages.

On the other hand, hybrid carbon and three-dimensional carbon nanomaterials are examples of modified carbon-based nanomaterial. Carbon-based materials can be easily functionalized with a variety of materials including biomolecules/proteins, nanoparticles, metal structures, and polymers, all of which absorb biomolecules or substances of choice in order to induce and control the behavior of stem cells. These hybrid materials were also shown to be effective in enhancement of stem cell growth and differentiation toward specific lineage, including bone cell generation, in 2D or 3D environments. Despite the fact that research on the use of carbon-based nanomaterials for bone tissue engineering is still in its early stages of development, there may be a brighter future for its advancement in biomedical applications, especially for the stem cell-based regenerative therapies, owing to the several advantages of carbon-based materials such as low cytotoxicity, biocompatibility and ease of functionalization with other types of biological components (e.g. DNAs/RNAs, proteins, biomolecules). Such technology could be also highly useful in healing various incurable disease/disorders, which still cannot be properly treated using existing medical technology.
